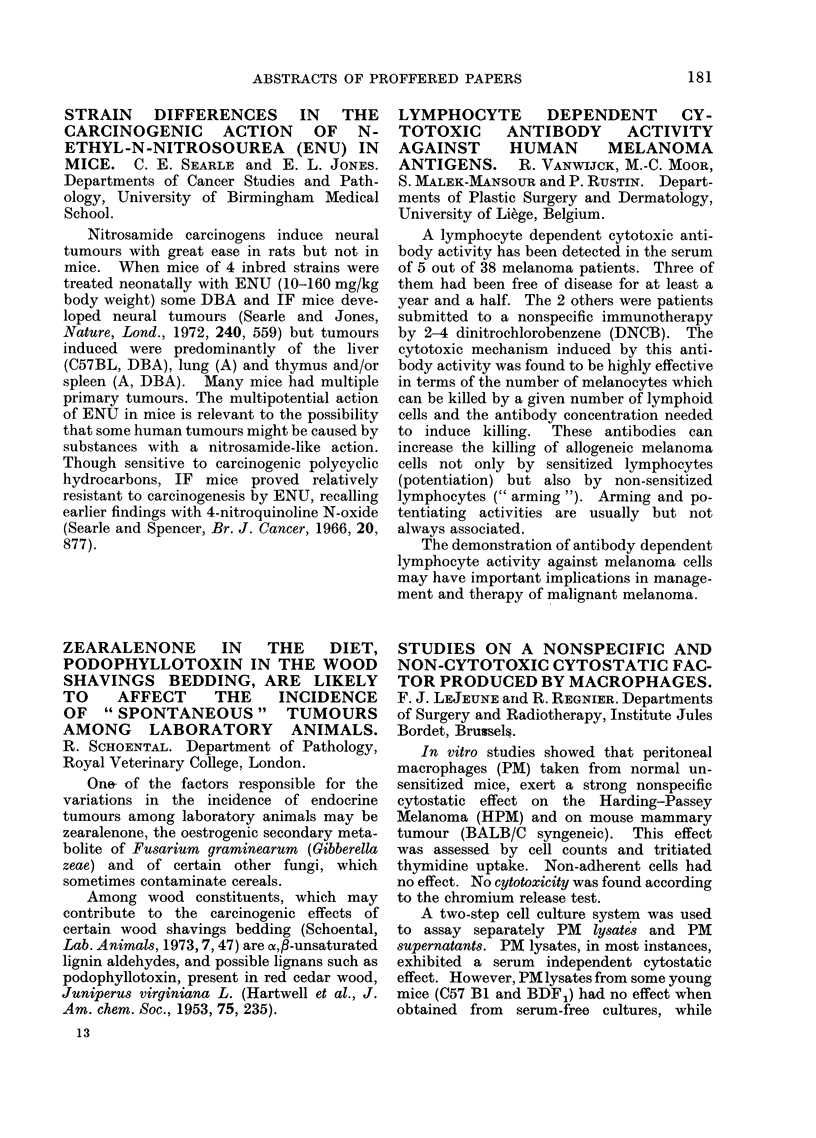# Proceedings: Strain differences in the carcinogenic action of N-ethyl-N-nitrosourea (ENU) in mice.

**DOI:** 10.1038/bjc.1974.158

**Published:** 1974-08

**Authors:** C. E. Searle, E. L. Jones


**STRAIN DIFFERENCES IN THE CARCINOGENIC ACTION OF N-ETHYL-N-NITROSOUREA (ENU) IN MICE.** C. E. SEARLE and E. L. JONES. Departments of Cancer Studies and Pathology, University of Birmingham Medical School.

Nitrosamide carcinogens induce neural tumours with great ease in rats but not in mice. When mice of 4 inbred strains were treated neonatally with ENU (10–160 mg/kg body weight) some DBA and IF mice developed neural tumours (Searle and Jones, *Nature, Lond.*, 1972, **240**, 559) but tumours induced were predominantly of the liver (C57BL, DBA), lung (A) and thymus and/or spleen (A, DBA). Many mice had multiple primary tumours. The multipotential action of ENU in mice is relevant to the possibility that some human tumours might be caused by substances with a nitrosamide-like action. Though sensitive to carcinogenic polycyclic hydrocarbons, IF mice proved relatively resistant to carcinogenesis by ENU, recalling earlier findings with 4-nitroquinoline N-oxide (Searle and Spencer, *Br. J. Cancer*, 1966, **20**, 877).